# Anti-Malarial Drug Artesunate Attenuates Experimental Allergic Asthma via Inhibition of the Phosphoinositide 3-Kinase/Akt Pathway

**DOI:** 10.1371/journal.pone.0020932

**Published:** 2011-06-09

**Authors:** Chang Cheng, W. Eugene Ho, Fera Y. Goh, Shou Ping Guan, Li Ren Kong, Wen-Qi Lai, Bernard P. Leung, W. S. Fred Wong

**Affiliations:** 1 Departments of Pharmacology, Yong Loo Lin School of Medicine, National University Health System, Singapore, Singapore; 2 Departments of Physiology, Yong Loo Lin School of Medicine, National University Health System, Singapore, Singapore; 3 Immunology Program, Life Science Institute; National University of Singapore, Singapore; University Hospital Freiburg, Germany

## Abstract

**Background:**

Phosphoinositide 3-kinase (PI3K)/Akt pathway is linked to the development of asthma. Anti-malarial drug artesunate is a semi-synthetic derivative of artemisinin, the principal active component of a medicinal plant *Artemisia annua*, and has been shown to inhibit PI3K/Akt activity. We hypothesized that artesunate may attenuate allergic asthma via inhibition of the PI3K/Akt signaling pathway.

**Methodology/Principal Findings:**

Female BALB/c mice sensitized and challenged with ovalbumin (OVA) developed airway inflammation. Bronchoalveolar lavage fluid was assessed for total and differential cell counts, and cytokine and chemokine levels. Lung tissues were examined for cell infiltration and mucus hypersecretion, and the expression of inflammatory biomarkers. Airway hyperresponsiveness was monitored by direct airway resistance analysis. Artesunate dose-dependently inhibited OVA-induced increases in total and eosinophil counts, IL-4, IL-5, IL-13 and eotaxin levels in bronchoalveolar lavage fluid. It attenuated OVA-induced lung tissue eosinophilia and airway mucus production, mRNA expression of E-selectin, IL-17, IL-33 and Muc5ac in lung tissues, and airway hyperresponsiveness to methacholine. In normal human bronchial epithelial cells, artesunate blocked epidermal growth factor-induced phosphorylation of Akt and its downstream substrates tuberin, p70S6 kinase and 4E-binding protein 1, and transactivation of NF-κB. Similarly, artesunate blocked the phosphorylation of Akt and its downstream substrates in lung tissues from OVA-challenged mice. Anti-inflammatory effect of artesunate was further confirmed in a house dust mite mouse asthma model.

**Conclusion/Significance:**

Artesunate ameliorates experimental allergic airway inflammation probably via negative regulation of PI3K/Akt pathway and the downstream NF-κB activity. These findings provide a novel therapeutic value for artesunate in the treatment of allergic asthma.

## Introduction

Allergic asthma is a chronic airway disorder characterized by airway inflammation, mucus hypersecretion, and airway hyperresponsiveness (AHR) [1]. Cumulative evidence revealed that these inflammatory responses are mediated by T-helper type 2 (Th2) cells together with mast cells, B cells and eosinophils, as well as a number of inflammatory cytokines and chemokines [1], [2]. IL-4 is imperative for B cell isotype switching for the synthesis of immunoglobulin (Ig)E. Allergen-induced crosslinking of IgE-bound high affinity IgE receptors (FcεRΙ) on the surface of mast cells leads to degranulation and activation of mast cells, and the release of inflammatory mediators like histamine, leukotrienes and cytokines, and immediate bronchoconstriction [3], [4]. IL-5 is vital for the growth, differentiation, recruitment, and survival of eosinophils which contribute to inflammation and even airway remodeling in asthma [5]. IL-13 plays a pivotal role in the effector phase of Th2 responses such as eosinophilic inflammation, mucus hypersecretion, AHR and airway remodeling [6]. In addition, chemokines such as RANTES (regulated on activation, normal T cells expressed and secreted) and eotaxin are crucial to the delivery of eosinophils to the airways [7]. Airway eosinophilia, together with Th2 cytokines IL-4, IL-5 and IL-13, may ultimately contribute to AHR in asthma [8].

Artesunateis a semi-synthetic derivative of artemisinin, a sesquiterpenetrioxane lactone isolated from the herb *Artemisia annua*. This medicinal plant has been used as a remedy for fevers and chills for centuries in China [9]. Artemisinin derivatives including artesunate are anti-malarial drugs effective for both uncomplicated and severe malaria [9], [10]. Besides, they have been shown to possess anti-cancer [11], [12], anti-viral [13], and anti-inflammatory [14], [15], [16], [17] activities. Artesunate has been reported to block the production of IL-1β, IL-6 and IL-8 from TNF-α-stimulated human rheumatoid arthritis fibroblast-like synoviocytes [14]. In addition, artesunate inhibits lipopolysaccharide-induced production of TNF-α, IL-6 and nitric oxide (NO), and expression of toll-like receptor 4 (TLR4) and TLR9 from macrophages [15], [16]. The exact molecular mechanism that mediates these anti-inflammatory effects by artesunate has not been unequivocally determined. There are some evidence pointing to the inhibition of nuclear factor NF-κB transcriptional activity by artesunate and other artemisinin derivatives [15], [16], [17]. More recently, artesunate has been found to possess strong inhibitory activity against the phosphoinositide 3-kinase (PI3K)/Akt signaling pathway [12], [13], [14].

As PI3K/Akt signaling pathway plays a critical role in the activation and immune responses of eosinophils, T and B lymphocytes, and mast cells [18], [19], [20], and genetic ablation of PI3Kδ or PI3Kγ isoform in mice has been shown to dampen Th2 immune responses, eosinophilic lung infiltration, and airway hyperresponsiveness and remodeling [21], [22], [23], we investigated the effects of artesunate on various aspects of ovalbumin (OVA)-induced Th2-mediated allergic airway inflammation in an *in vivo* mouse asthma model and explored the anti-inflammatory mechanism of action of artesunate. We also verified the anti-inflammatory effect of artesunate in a house dust mite mouse asthma model. Our results clearly indicate that artesunate attenuates allergic airway inflammation and it is likely mediated through inhibition of the PI3K/Akt signaling pathway.

## Results

### Artesunate suppresses OVA-induced inflammatory cell recruitment and mucus production

**Figure 1:**
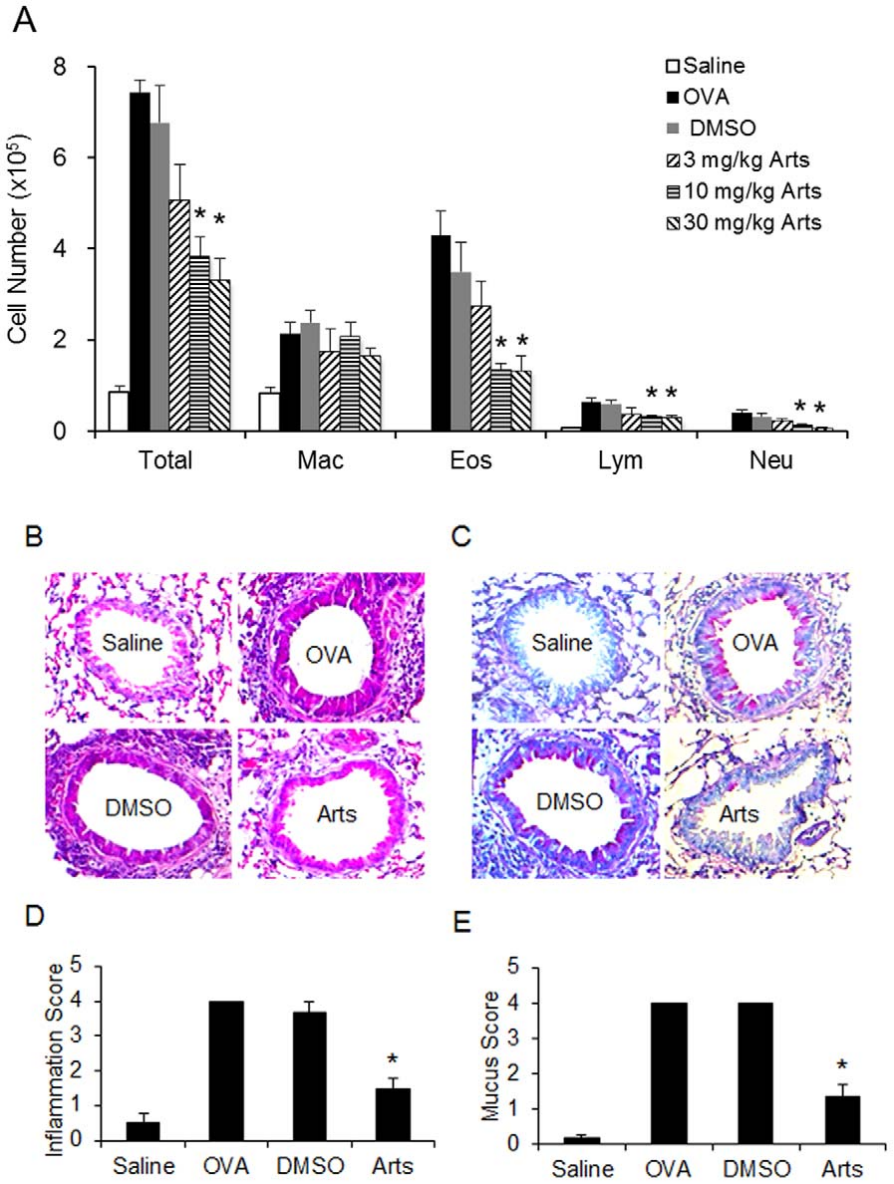
Effects of artesunate on OVA-induced inflammatory cell recruitment and mucus hypersecretion. (A) Inflammatory cell counts in BAL fluid obtained from sensitized mice 24 hours after the last saline aerosol (n = 7 mice) or OVA aerosol (n = 9 mice) challenge. Artesunate dose-dependently reduced OVA-induced inflammatory cell counts in BAL fluid from sensitized mice 24 hours after the last OVA aerosol challenge (DMSO, n = 9; 3 mg/kg, n = 7; 10 mg/kg, n = 9; and 30 mg/kg, n = 10). Differential cell counts were performed on a minimum of 500 cells to identify eosinophil (Eos), macrophage (Mac), neutrophil (Neu), and lymphocyte (Lym). Histological sections of lung tissue eosinophilia (B, magnification×200) and mucus secretion (C, magnification×200) 24 hours after the last challenge of saline aerosol, OVA aerosol, OVA aerosol plus DMSO, or OVA aerosol plus 30 mg/kg artesunate were evaluated. Quantitative analyses of inflammatory cell infiltration (D) and mucus production (E) in lung sections were performed as previously described [56]. Scoring of inflammatory cells and goblet cells was performed in at least 3 different fields for each lung section. Mean scores were obtained from 4 animals. *Significant difference from DMSO control, p<0.05.

Bronchoalveolar lavage (BAL) fluid was collected 24 hours after the last OVA or saline aerosol challenge, and total and differential cell counts were performed. OVA inhalation markedly increased total cell and eosinophil counts, and slightly yet significantly (p<0.05) increased macrophage, lymphocyte and neutrophil counts, as compared with saline aerosol control. Artesunate (3, 10 and 30 mg/kg) drastically decreased the total cell and eosinophil counts in BAL fluid in a dose-dependent manner as compared with the DMSO vehicle control (**Figure 1A**). We have conducted flow cytometric analysis of peripheral blood leukocytes obtained from saline-challenged, OVA-challenged, vehicle control, and artesunate-treated mice. Similar percentages of CD3+, CD4+, CD8+ T cells, B cells (B220), NK cells (NK 1.1), neutrophils and monocytes were observed in all mice (data not shown). Hence, artesunate-induced reduction of eosinophil and lymphocyte pulmonary recruitment is unlikely due to any potential nonspecific cytotoxic effects of the drug.

Lung tissue was also collected 24 hours after the last OVA or saline aerosol challenge. OVA aerosol challenge induced marked infiltration of inflammatory cells into the peribronchiolar and perivascular connective tissues as compared with saline aerosol challenge. Artesunate (30 mg/kg) markedly diminished the eosinophil-rich leukocyte infiltration as compared with DMSO control (**Figure 1B and 1D**). On the other hand, OVA-challenged mice, but not saline-challenged mice, developed marked goblet cell hyperplasia and mucus hypersecretion in the bronchi. OVA-induced mucus hypersecretion was significantly halted by artesunate (30 mg/kg) (**Figure 1C and 1E**).

### Artesunate reduces OVA-induced BAL fluid Th2 cytokine levels

**Figure 2:**
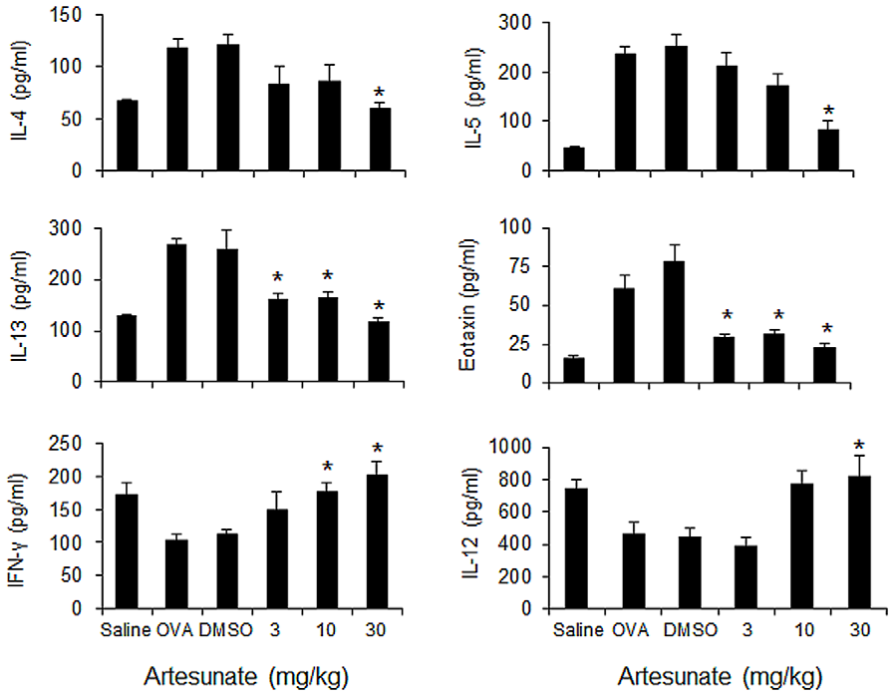
Effects of artesunate on OVA-induced BAL fluid cytokine and chemokine levels. BAL fluids were collected 24 hours after the last OVA aerosol challenge. Levels of IL-4, IL-5, IL-12, IL-13, eotaxin and IFN-γ were analyzed using ELISA (n = 6–9 mice). Lower limits of detection were as follows: IL-4 and IL-5 at 4 pg/ml; IL-12, IL-13 and IFN-γ at 15.6 pg/ml; and eotaxin at 2 pg/ml. Values shown are the mean ± SEM. *Significant difference from DMSO control, p<0.05.

OVA inhalation in sensitized mice caused a notable increase in IL-4, IL-5, IL-13 and eotaxin levels in BAL fluid as compared with saline aerosol control (**Figure 2**). In contrast, BAL fluid level of IFN-γ and IL-12, two Th1 cytokines, dropped slightly in OVA-challenged mice. Artesunate drastically reduced IL-13 and eoxtain, and to a lesser extent, IL-4 and IL-5 levels in BAL fluid in a dose-dependent manner as compared with DMSO control (**Figure 2**). Noticeably, artesunate at 10 and 30 mg/kg could up-regulated IFN-γ and IL-12 levels in BAL fluid levels similar to those in saline control mice. This finding implies that artesunate is able to modify the Th2-predominant immune activity in our OVA-induced mouse asthma model. Alternatively, the increase in IL-12 BAL fluid level in artesunate-treated mice may be due to enhanced IL-12 production by dendritic cells upon inhibition of PI3K [24]. The exact mechanism that mediates up-regulation of IFN-γ and IL-12 BAL fluid levels remains to be determined.

### Artesunate suppresses OVA-specific lymphocyte responses *in vitro*

**Figure 3:**
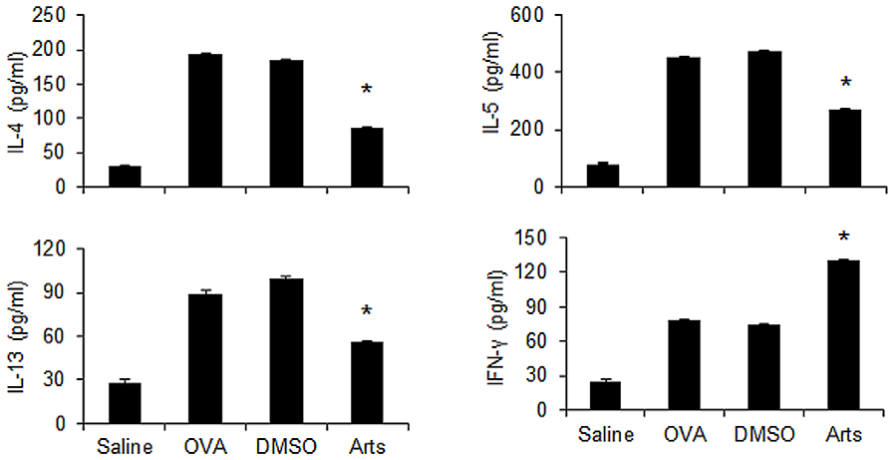
Effects of artesunate on OVA-specific lymphocyte response *in vitro*. Thoracic lymph nodes cells (n = 4 mice) were harvested from mice 24 hours after the last OVA or saline aerosol challenge, and cultured for 72 hours with medium alone or OVA (200 µg/ml). The levels of IL-4, IL-5, IL-13 and IFN-γ in culture supernatant were determined using ELISA. Values shown are the mean ± SEM of triplicate cultures of pooled lymph node cell suspensions. *Significant difference from DMSO control, p<0.05.

To assess whether artesunate treatment could suppress lymphocyte cytokine production, we examined OVA-specific immune responses in thoracic lymph node cultures. The *in vitro* OVA-specific production of IL-4, IL-5 and IL-13 was markedly higher in lymphocytes isolated from OVA-challenged mice than those from saline-challenged mice (**Figure 3**). Artesunate (30 mg/kg) pretreatment significantly (p<0.05) lowered the levels of IL-4, IL-5 and IL-13. In contrast, *in vitro* OVA-specific IFN-γ production was found to be elevated in mice treated with artesunate (30 mg/kg). The observed immune modulation by artesunate *in vitro* was OVA-specific because Con A-induced production of IL-4, IL-5, IL-13 and IFN-γ in parallel cultures was not affected (data not shown).

### Artesunate reduces OVA-induced airway hyperresponsiveness in mice

**Figure 4:**
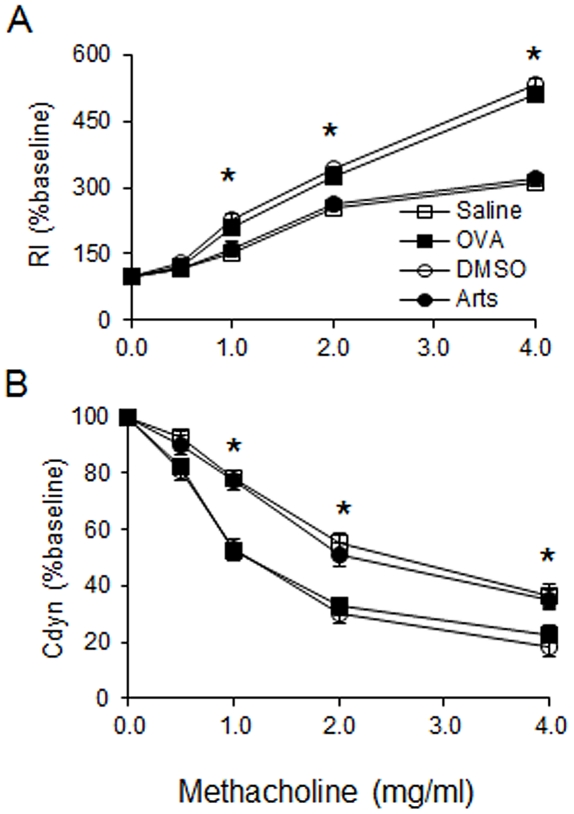
Effects of artesunate on OVA-induced AHR. Airway responsiveness of mechanically ventilated mice in response to aerosolized methacholine was measured 24 hours after the last saline aerosol or OVA aerosol with pretreatment of either DMSO or 30 mg/kg artesunate. AHR is expressed as percentage change from the baseline level of (A) lung resistance (Rl, n = 6 mice) and (B) dynamic compliance (Cdyn, n = 6 mice). Rl is defined as the pressure driving respiration divided by flow. Cdyn refers to the distensibility of the lung and is defined as the change in volume of the lung produced by a change in pressure across the lung. *Significant difference from DMSO control, p<0.05.

To investigate the effect of artesunate on airway hyperresponsiveness (AHR) in response to increasing concentrations of methacholine, we measured both RI and Cdyn in mechanically ventilated mice. Rl is defined as the pressure driving respiration divided by flow. Cdyn refers to the distensibility of the lung and is defined as the change in volume of the lung produced by a change in pressure across the lung. OVA-challenged mice developed AHR which is typically reflected by high RI and low Cdyn (**Figure 4**). Artesunate (30 mg/kg) dramatically reduced RI and restored Cdyn in OVA-challenged mice in response to methacholine aerosol, suggesting that immune-mediated airway pathology *in vivo* was modified.

### Artesunate inhibits OVA-induced inflammatory gene expression and PI3K/Akt activation in allergic airway inflammation

**Figure 5:**
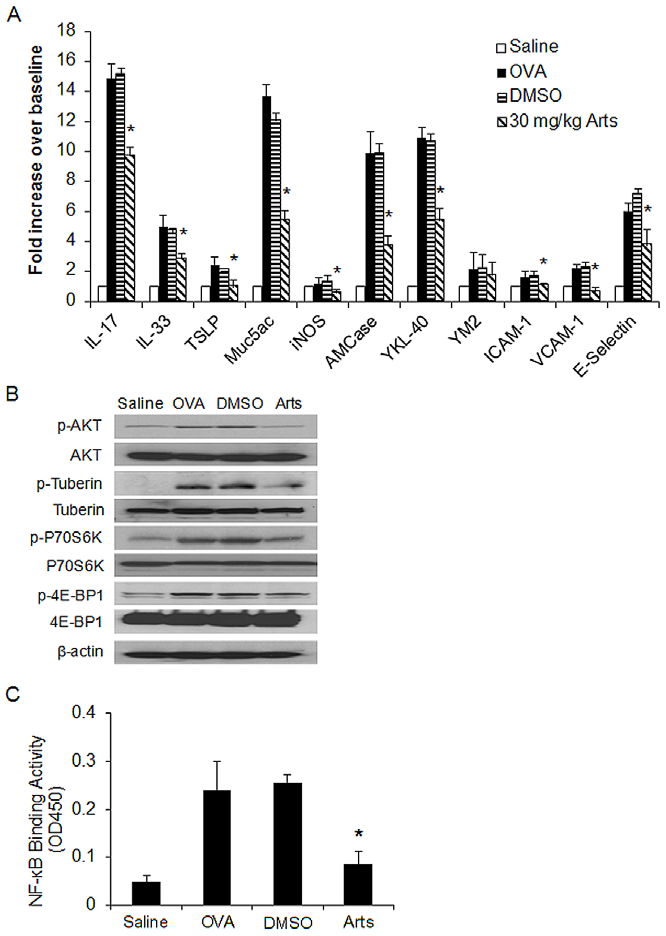
Effects of artesunate on OVA-induced inflammatory gene expression, PI3K/Akt activation and NF-κB DNA-binding activity in allergic airway inflammation. (A) Lung tissues were collected 24 hours after the last OVA aerosol challenge. Total mRNA was extracted using TriZol reagent and the quantitative real time PCR was performed. All reactions were run in triplicate and three independent experiments for each target. The relative quantity of target gene expression was automatically normalized by GADPH as an internal control and values shown were the ratios of various treatments to saline group. (B) Immunoblotting of Akt, tuberin, p70S6K and 4E-BP1 in protein extracts of lung tissues isolated from mice 24 hours after the last saline aerosol or OVA aerosol challenge pretreated with either DMSO or 30 mg/kg artesunate. β-actin was used as an internal control. The experiments were repeated for three times (n = 3 mice) with similar pattern of results. (C) Nuclear p65 DNA-binding activity was determined using a TransAM™ p65 transcription factor ELISA kit. Values shown are the mean ± SEM of four separate experiments. *Significant difference from DMSO control, p<0.05.

OVA aerosol challenge markedly up-regulated lung mRNA level of Muc5ac, which is essential for mucus hypersecretion [25]; inducible nitric oxide synthase (iNOS), the enzyme responsible for nitric oxide (NO) production in allergic airway inflammation [26]; thymic stromal lymphopoietin (TSLP), a cytokine key to the initiation of Th2 immune response [27]; IL-17 and IL-33, two effector cytokines that have recently been shown essential for airway inflammation and remodeling [28], [29]; of chitinase family members including acidic mammalian chitinase (AMCase), Ym2 and YKL-40, which have recently been shown to play critical roles in airway inflammation and remodeling [30], [31], [32]; and of adhesion molecules such as ICAM-1, VCAM-1 and E-selectin, which are pivotal for pulmonary recruitment of inflammatory cells like eosinophils and lymphoctyes [7], [33]. Pretreatment with artesunate (30 mg/kg) demonstrated strong suppression of Muc5ac, iNOS, TSLP, IL-17, IL-33, AMCase, Ym-2, YKL-40, ICAM-1, VCAM-1 and E-selectin, in the allergic airways (**Figure 5A**).

To verify that the anti-inflammatory mechanism of action by artesunate in OVA-challenged mice was mediated through the inhibition of the PI3K/Akt signaling pathway, we examined the phosphorylation cascade of Akt, tuberin, p70 ribosomal S6 kinase (p70S6K) and eukaryotic initiation factor 4E-binding protein 1 (4E-BP1) in lung tissues obtained 24 hours after the last OVA or saline aerosol challenge. OVA challenge markedly raised the phosphorylation state of Akt(ser^473^), tuberin(thr^1462^), p70S6K(thr^389^) and 4E-BP1(ser^65^) as compared with saline aerosol control (**Figure 5B**). Artesunate (30 mg/kg) markedly reduced the phsophorylation of Akt, tuberin, p70S6K and 4E-BP1 to the basal levels. Besides, PI3K/Akt pathway activation has been shown to promote NF-κB DNA binding activity [34]. Artesunate significantly suppressed OVA-induced NF-κB transactivation in lung tissues to basal level (**Figure 5C**). Our findings suggest that artesunate may exert its anti-inflammatory actions via inhibition of PI3K/Akt pathway.

### Artesunate inhibits EGF-induced PI3K/Akt activation in primary human bronchial epithelial cells

**Figure 6:**
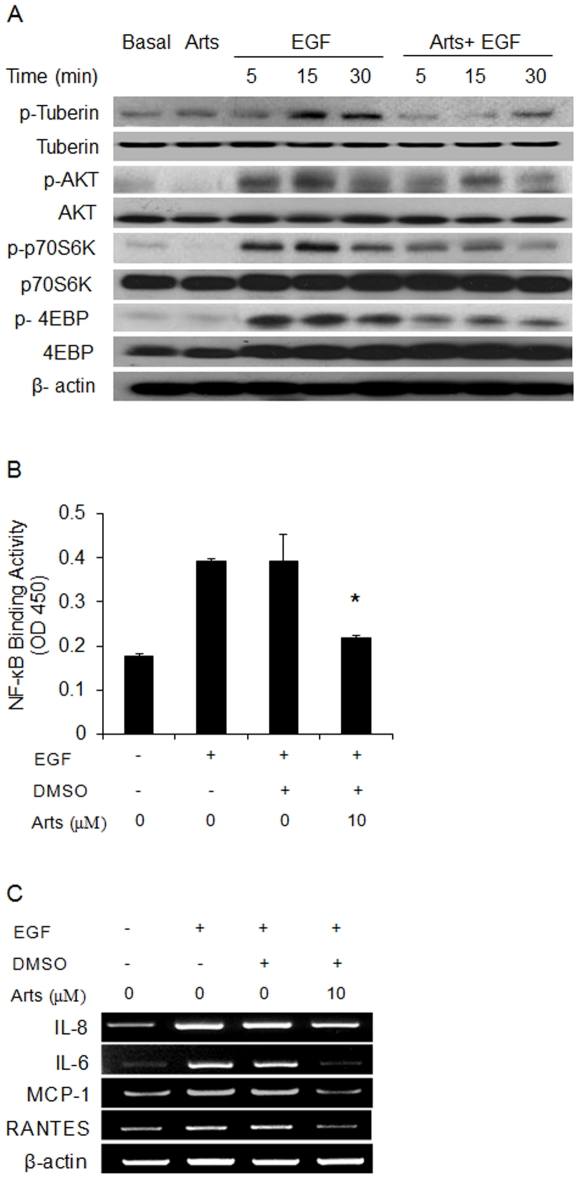
Effects of artesunate on EGF stimulation of normal human bronchial epithelial cells. (A) Epithelial cells were stimulated with 100 ng/ml EGF in the presence and absence of 10 µM artesunate for 5, 15 and 30 minutes before total proteins were extracted for subsequent immunoblotting analysis. β-actin was used as an internal control. (B) DNA-binding activity of p65 NF-κB in nuclear extracts of epithelial cells stimulated with EGF for 30 minutes in the presence and absence of 10 µM artesunate was determined using a TransAM™ p65 transcription factor ELISA kit. (C) Epithelial cells were stimulated with 100 ng/ml EGF in the presence and absence of 10 µM artesunate for 12 hours before total mRNA was extracted using TriZol reagent. PCR products were separated in a 2% agarose gel and visualized under UV light. β-actin was used as an internal control. Representative gels from 4 separate experiments with similar pattern of results. Values shown are the mean ± SEM of three separate experiments. *Significant difference from DMSO control, p<0.05.

To further explore anti-inflammatory mechanisms of action of artesunate in a relevant human airway cell type, we studied the effects of artesunate on epidermal growth factor (EGF)-induced activation of PI3K/Akt signaling pathway and cytokine mRNA expression in normal human bronchial epithelial cells. EGF plays a critical role in asthma [35], [36] and is a potent stimulator of human airway epithelial cells [37]. EGF induced a rapid phosphorylation of Akt, tuberin, p70S6K and 4E-BP1 (**Figure 6A**). This was accompanied with the up-regulation of NF-κB DNA binding activity (**Figure 6B**). Artesunate markedly inhibited the EGF-induced phosphorylation of Akt, tuberin, p70S6K and 4E-BP1, and NF-κB transactivation. Furthermore, artesunate noticeably blocked EGF-induced up-regulation of IL-6, IL-8, monocyte chemoattractant protein-1 (MCP-1) and RANTES mRNA expression in human bronchial epithelial cells (**Figure 6C**). MTT assay was conducted to examine potential cytotoxicity of artesunate on normal human bronchial epithelial cells. No cytotoxicity on bronchial epithelial cells was observed with artesunate drug concentrations (0.1–100 µM) used in the *in vitro* experiments (data not shown).

### Artesunate suppresses airway inflammation in a house dust mite extract-induced asthma model

**Figure 7:**
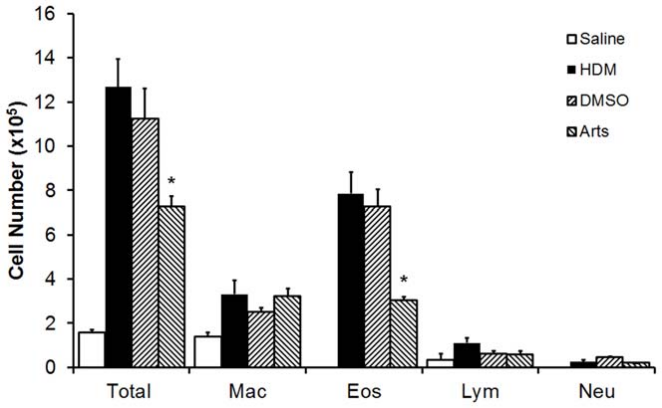
Effects of artesunate on house dust mite extracts-induced pulmonary inflammatory cell recruitment. Inflammatory cell counts in BAL fluid obtained from mice 72 hours after the last house dust mitechallenge (n = 6 mice) markedly increased as compared to saline control (n = 4 mice). Artesunate (30 mg/kg, n = 6 mice) significantly suppressed house dust mite-induced increases in BAL fluid total cell and eosinophil counts as compared with vehicle control (DMSO, n = 5). Differential cell counts were performed on a minimum of 500 cells to identify (Eos), macrophage (Mac), neutrophil (NEU), and lymphocyte (Lym). *Significant difference from DMSO control, p<0.05.

To validate the anti-inflammatory effects of artesunate in airway inflammation more comparable to human situation, we have developed a house dust mite mouse asthma model as described previously [38], [39]. Intratracheal administration of house dust mite extract (100 µg per mouse) on day 0, day 7 and day 14 resulted a notable increase in BAL fluid total cell and eosinophil counts, as compare with saline control. Pre-treatment with artesunate (30 mg/kg) significantly (p<0.05) suppressed the total and eosinophil counts in BAL fluid as compared with the DMSO vehicle control (**Figure 7**).

## Discussion

PI3K is a family of lipid kinases comprising of 8 isoforms, of which p110δ and p110γ PI3Ks are enriched in leukocytes and have been shown to play a critical role in the activation, proliferation, differentiation and migration of T and B lymphocytes, mast cells and eosinophils [18]–[20]. Indeed, genetic ablation of p110δ or p110γ PI3K in mice has been shown to dampen Th2 immune responses, eosinophilic lung infiltration, and airway hyperresponsiveness and remodeling [21]–[23]. Therapeutic strategies targeted at the PI3K/Akt signaling pathway such as using adenovirus-carrying phosphatase and tensin homologue deleted on chromosome ten (PTEN) cDNA [40], dominant-negative class IA PI3K-TAT fusion protein [41], and PI3K non-selective and isoform-selective small molecule inhibitors [42], [43], [44] have demonstrated beneficial effects in experimental asthma models. Our findings reveal for the first time significant inhibition of Akt(ser^473^) phosphorylation and of its downstream kinases by artesunate in both OVA-challenged lungs *in vivo* and EGF-stimulated normal human bronchial epithelial cells *in vitro*. Taken together, we have established that anti-malarial drug artesunate, a semi-synthetic derivative of artemisinin isolated from the herb *Artemisia annua*, can effectively suppress various aspects of OVA-induced Th2-mediated allergic airway inflammation in mice via inhibition of the PI3K/Akt signaling pathway.

Th2 cytokines play an essential role in the pathogenesis of the allergic airway inflammation [1], [2]. IL-4, IL-5 and IL-13 can be produced by various lung resident cells such as bronchial epithelial cells, mast cells and alveolar macrophages as well as infiltrated inflammatory cells such as lymphocytes and eosinophils. Our present results show that artesunate significantly reduced the level of IL-4, IL-5, IL-13 and eotaxin in BAL fluids from OVA-challenged mice. They are in line with studies using mice deficient in p110δ or p110γ PI3K showing a major drop in Th2 cytokines and chemokines upon allergen sensitization and challenge [21], [22]. Artesunate was found to suppress OVA-induced phosphorylation of Akt and its downstream signaling molecules tuberin, p70S6K and 4EBP1, and transactivation of NF-κB in lung samples. Cumulative evidence has shown that inhibitors targeting at the p70S6K and NF-κB could dampen Th2 immune responses in animal models of asthma [45], [46]. Therefore, the observed reduction of IL-4, IL-5, IL-13 and eotaxin levels in BAL fluid from artesunate-treated mice may be due to inhibition of PI3K/Akt and its downstream kinases in the inflammatory and airway resident cells. Furthermore, our *in vitro* study using direct OVA stimulation of lymph node cells isolated from artesunate-treated mice produced markedly lower level of IL-4, IL-5 and IL-13 together with higher IFN-γ level as compared with DMSO-treated mice. These data show that the anti-inflammatory effect of artesunate is at least in part mediated through a direct suppressive action on T lymphocytes.

Eosinophils play a central role in the pathogenesis of allergic inflammation [5], [7]. Our present findings showed that artesunate prevented OVA-induced inflammatory cell infiltration into the airways as shown by a significant drop in total cell counts and eosinophil and lymphocyte counts in BAL fluid, and in tissue eosinophilia in lung sections. To validate the anti-inflammatory effects of artesunate in a more clinically relevant asthma model, we also observed a major reduction of total cell and eosinophil counts in BAL fluid collected from house dust mite extract-challengedmice. Leukocyte transmigration into the airways is orchestrated by cytokines like IL-4, IL-5 and IL-13, and coordinated by specific chemokines like eotaxin and RANTES in combination with adhesion molecules such as intercellular adhesion molecule-1 (ICAM-1), vascular cell adhesion molecule-1 (VCAM-1) and E-selectin [7], [33]. Cytokine receptor activation by IL-4, IL-5 and IL-13 induces PI3K/Akt signaling cascade [47], [48], and IL-4 and IL-13 are potent inducers of eotaxin and RANTES expression in human bronchial epithelial cells [49]. PI3Kγ plays an imperative role in mediating eotaxin-induced eosinophil chemotaxis *in vitro* [18]. Inhibition of class IA PI3K in eosinophil has been shown to block IL-5-induced β_2_-integrin adhesion of human eosinophils to ICAM-1 [48]. In addition, selective PI3Kδ inhibition in mice suppressed aeroallergen-induced VCAM-1 and ICAM-1 expression in lungs [43]. We have demonstrated that artesunate strongly suppressed ICAM-1, VCAM-1 and E-selectin mRNA expression and eotaxin production in OVA-challenged lungs, and IL-8, RANTES and MCP-1 mRNA expression in EGF-stimulated normal human bronchial epithelial cells. Taken together, the observed reduction in airway eosinophilia by artesunate may be a result of combined inhibitory effects on IL-4, IL-5, IL-13, eotaxin and RANTES production, and on adhesion molecule expression, secondary to inhibition of PI3K/Akt.

We have also demonstrated a dramatic reduction in airway mucus production in artesunate-treated mice as compared with DMSO control. Cumulative evidence indicates that IL-4, IL-5, IL-6, IL-13, IL-17 and IL-33 induce goblet cell hyperplasia and mucin production in mice [25], [28], [29], [37]. Mice deficient in p110δ or p110γ PI3K had impaired mucus production in response to aeroallergen challenge [21], [22]. Muc5ac gene expression is also dependent on the transcriptional activity of NF-κB [25], [50]. We also observed a substantial drop in Muc5ac mRNA expression by artesunate in OVA-challenged lungs. As such, the marked decrease in mucus production in the lungs of artesunate-treated mice may be attributable to a significant reduction of Th2 cytokine levels, together with inhibition of NF-κB transactivation in airway epithelium.

A family of chitinase proteins including AMCase, Ym1, Ym2 and YKL-40 has recently been found to be markedly elevated in allergic airway inflammation in human and in mouse asthma models [30], [31], [32]. AMCase level is increased in a mouse asthma model and in asthmatic subjects. When given intratracheally, IL-13 elevates Ym1 and Ym2 levels in BAL fluid from mice *in vivo* [51]. Besides, YKL-40 serum level correlates positively with asthma severity, airway remodeling and deterioration of pulmonary function in asthmatic subjects [32]. Overall, chitinases may play a role in airway inflammation and remodeling. Our data show that artesunate markedly down-regulated AMCase, Ym2 and YKL-40 mRNA expression in the lungs of OVA-challenged mice. These may be a consequence of the major drop in IL-4 and IL-13 levels in the airways with artesunate treatment and may contribute to the diminished pulmonary eosinophilia.

Patients with bronchial asthma produce higher level of exhaled NO as compared with healthy controls, and the NO level may reflect the severity of asthma [52]. It appears that increased exhaled NO is associated with increased iNOS expression in the lung epithelium of asthma patients [53]. IL-13 has been shown to induce iNOS expression in normal human bronchial epithelial cells leading to elevate NO production [26]. PI3K plays an important role in the dimerization of iNOS for the NO production [54]. In addition, iNOS gene expression is regulated by the NF-κBtranscriptional activity [50]. Our results show that artesunate markedly suppressed the OVA-induced iNOS expression in the lungs, which may be due to inhibition of PI3K/Akt pathway and of the downstream NF-κB activity, and the reduced level of IL-13 in the allergic airways.

Activation of PI3K/Akt and its downstream molecules tuberin, p70S6K and 4E-BP1 lead to airway smooth muscle hypertrophy and hyperplasia [55], [56]. More recently, p110δ PI3K has been shown to mediate IL-13-induced mouse tracheal smooth muscle hyperreactivity to methacholine [57]. We report here that artesunate significantly inhibited OVA-induced AHR to increasing concentrations of methacholine. Thus, the observed reduction of AHR by artesunate may be associated with the reduction in Th2 cytokine production, tissue eosinophilia, and airway smooth muscle contractile machinery via inhibition of PI3K/Akt signaling pathway.

We report here for the first time that the anti-malarial agent artesunate effectively reduced OVA-induced inflammatory cell recruitment into BAL fluid, IL-4, IL-5, IL-13 and eotaxin production, pulmonary eosinophilia, mucus hypersecretion and AHR in a mouse asthma model potentially via inhibition of the PI3K/Akt signaling pathway. Artesunate is a clinically effective drug for both uncomplicated and severe malaria [9], [10], [11]. Our present findings support a novel therapeutic use of artesunate in the treatment of asthma.

## Materials and Methods

### Animalsand ethics statement

All animal experiments were performed according to the Institutional guidelines for Animal Care and Use Committee of the National University of Singapore. Female BALB/c mice, 6 to 8 weeks old (Interfauna, East Yorkshire, UK), were sensitized and challenged with OVA as described [58]. Briefly, mice were sensitized by i.p. injections of 20 µg OVA and 4 mg Al(OH)_3_ suspended in 0.1 ml saline on days 0 and 14. On days 22, 23 and 24, mice were challenged with 1% OVA aerosol for 30 minutes. Artesunate (3, 10, and 30 mg/kg; Sigma, St. Louis, MO) or vehicle (DMSO) in 0.1 ml saline was given by i.p. injections 1 hour before each OVA aerosol challenge. Saline aerosol was used as a negative control. For the development of house dust mite mouse asthma model, female BALB/c mice were anaesthetized using isoflurane (Halocarbon Products Corporation, River Edge, NJ, USA) and then administered through intratracheal route with either 40 µl of *Dermatophagoidespteronyssinus* extracts (100 µg, Greer Laboratories, Lenoir, NC, USA) or saline as a negative control on days 0, 7 and 14 as described [38], [39]. Artesunate (30 mg/kg) or vehicle in 0.1 ml saline was given by i.p on days 6, 7 and 8, and days 13, 14 and 15. Mice were sacrificedon day 17 and BAL fluid was collected for total and differential cell countsas described below.

### BAL fluid and serum analysis

Mice were anesthetized 24 hours after the last aerosol challenge and BAL was performed as described [58]. Briefly, tracheotomy was performed, and a cannula was inserted into the trachea. Ice-cold PBS (0.5 ml×3) was instilled into the lungs, and BAL fluid was collected. BAL fluid total and differential cell counts, and cytokine and chemokine levels were determined as described [58]. IL-4, IL-5 and IL-12 ELISA were obtained from BD PharMingen (San Diego, CA, USA). Eotaxin, IL-13 and IFN-γ ELISA were purchased from R&D Systems (Minneapolis, MN, USA).

### Histologic analysis

Lungs were fixed in 10% neutral formalin, paraffinized, cut into 5-µm sections, and stained with hematoxylin and eosin (H&E) for examining cell infiltration and with periodic acid-Schiff stain (PAS) for measuring mucus production. Quantitative analyses of cell infiltration and mucus production were performed blind as previously described [56].

### Measurements of AHR

Mice were anesthetized and tracheotomy was performed as described [58]. The trachea was intubated with a cannula that was connected to the pneumotach, ventilator and nebulizer. Lung resistance (Rl) and dynamic compliance (Cdyn) in response to increasing concentrations of nebulized mechacholine (0.5–8.0 mg/ml) were recorded using FinePointe™ data acquisition and analysis software (Buxco, Wilmington, NC, USA) as described [58]. Results are expressed as a percentage of the respective basal values in response to phosphate-buffered saline (PBS).

### Cell cultures

To determine the effects of artesunate on OVA-specific immune responses in lymphocytes, thoracic lymph node cells were prepared as described [58]. Cells were exposed to 200 µg/ml OVA for 72 hours. Concanavalin A (Con A, 10 µg/ml) was used as a positive control. Supernatants from parallel triplicate cultures were analyzed for cytokine levels by ELISA. Normal human bronchial epithelial cells (Lonza, Basel, Switzerland) were cultured in optimized bronchial epithelial bulletkit medium with supplements. Cells of passage numbers 3–6 were pretreated with 10 µM artesunate or vehicle (0.01% DMSO) 2 hours before stimulation with 100 ng/ml epidermal growth factor (EGF). Total and nuclear proteins, and mRNA were extracted from cells at specified time intervals.

### Immunoblotting, mRNA expression and NF-κB DNA-binding

Lung and cell culture protein lysates (20 mg per lane) were separated by 10% SDS-PAGE and immunoblots were developed as described [58]. Immunoblots were probed with anti-Akt, anti-phospho-Akt (Ser^473^), anti-tuberin, anti-phospho-tuberin (Ser^1462^), anti-p70S6K, anti-phospho-p70S6K (Ser^389^), anti-4E-BP1, anti-phospho-4E-BP1 (Ser^65^), and anti-β-actin antibodies (Cell Signaling Technology, Beverly, MA). Primers for inflammatory genes are shown in Table 1 and mRNA expression was analyzed by quantitative real time PCR (ABI 750 Cycler, Applied Biosystems, Carlsbad, CA, USA). Nuclear proteins were analyzed for NF-κB DNA-binding activity using TransAM NF-κB transcription factor assay kit (Active Motif, Carlsbad, CA).

**Table 1:** Primer sets for reverse transcriptase-polymerase chain reaction analysis.

Targets	Sequences	
	Forward	Reverse
m AMCase	5′-TGGGTTCTGGGCCTACTATG-3′	5′-GCTTGACAATGCTGCTGGTA-3′
m Ym2	5′-CAGAACCGTCAGACATTCATTA-3′	5′-ATGGTCCTTCCAGTAGGTAATA-3′
m YKL-40	5′-GTACAAGCTGGTCTGCTACT-3′	5′-GTTGGAGGCAATCTCGGAAA-3′
m ICAM-1	5′-CATCGGGGTGGTGAAGTCTGT-3′	5′-TGTGGGGGAAGTGTGGTC-3′
m VCAM-1	5′-CAAGGGTGACCAGCTCATGAA-3′	5′-TGTGCAGCCACCTGAGATCC-3′
m E-selectin	5′-AACGCCAGAACAACAATTCC-3′	5′-TGAATTGCCACCAGATGTGT-3′
m Muc5ac	5′-GAGTGACATTGCAGGAAGCA-3′	5′-CAGAGGACAGGAAGGTGAGC-3′
m iNOS	5′-GTCAACTGCAAGAGAACGGAGAC-3′	5′-GAGCTCCTCCAGACGGGTAGGCTTG-3′
m TSLP	5′-CGACAGCATGGTTCTTCTCA-3′	5′-CGACAGCATGGTTCTTCTCA-3′
m IL-17A	5′-CCGCAATGAAGACCCTGATAGA-3′	5′-CAGCATCTTCTCGACCCTGAAA-3′
m IL-33	5′-GATGGGAAGAAGGTGATGGGTG-3′	5′-TTGTGAAGGACGAAGAAGGC-3′
h IL-6	5′-CAGGAGAAGATTCCAAAGAT-3′	5′-ACTGGTTCTGTGCCTGCAGC-3′
h IL-8	5′-ATGACTTCCAAGCTGGCCGTGGCT-3′	5′-TCTCAGCCCTCTTCAAAAACTTCTC-3′
h RANTES	5′-ATGAAGGTCTCCGCGGCACGCCT-3′	5′-CTAGCTCATCTCCAAAGAGTTG-3′
h MCP-1	5′-GATCTCAGTGCAGAGGCTCG-3′	5′-TGCTTGTCCAGGTGGTCCAT-3′
h/m β-Actin	5′-TCATGAAGTGTGACGTTGACATCCGT-3′	5′-CCTAGAAGCATTTGCGGTGCACGATG-3′

### Statistical analysis

Data are presented as means ± SEM. One-way *ANOVA* followed by Dunnett's test was used to determine significant differences between treatment groups. Significant levels were set at p<0.05.
